# Multielemental Analysis Associated with Chemometric Techniques for Geographical Origin Discrimination of Tea Leaves (*Camelia sinensis*) in Guizhou Province, SW China

**DOI:** 10.3390/molecules23113013

**Published:** 2018-11-18

**Authors:** Jian Zhang, Ruidong Yang, Rong Chen, Yuncong C. Li, Yishu Peng, Chunlin Liu

**Affiliations:** 1 College of Resource and Environmental Engineering, Guizhou University, Guiyang 550025, China; jzhanggzdxhjkx@163.com (J.Z.); pengys520@126.com (Y.P.); gzdxzhxylcl@163.com (C.L.); 2 College of Mining, Guizhou University, Guiyang 550025, China; rchengzu@163.com; 3 Department of Soil and Water Sciences, Tropical Research and Education Center, IFAS, University of Florida, Homestead, FL 33031, USA; yunli@ufl.edu

**Keywords:** tea leaves, multielement, ICP-MS, chemometrics, geographical origin discrimination

## Abstract

This study aimed to construct objective and accurate geographical discriminant models for tea leaves based on multielement concentrations in combination with chemometrics tools. Forty mineral elements in 87 tea samples from three growing regions in Guizhou Province (China), namely Meitan and Fenggang (MTFG), Anshun (AS) and Leishan (LS) were analyzed. Chemometrics evaluations were conducted using a one-way analysis of variance (ANOVA), principal component analysis (PCA), linear discriminant analysis (LDA), and orthogonal partial least squares discriminant analysis (OPLS-DA). The results showed that the concentrations of the 28 elements were significantly different among the three regions (*p* < 0.05). The correct classification rates for the 87 tea samples were 98.9% for LDA and 100% for OPLS-DA. The variable importance in the projection (VIP) values ranged between 1.01–1.73 for 11 elements (Sb, Pb, K, As, S, Bi, U, P, Ca, Na, and Cr), which can be used as important indicators for geographical origin identification of tea samples. In conclusion, multielement analysis coupled with chemometrics can be useful for geographical origin identification of tea leaves.

## 1. Introduction

The identification of the geographical origin of tea leaves (*Camelia sinensis*) is remarkably important in current globalized trade because it is directly related to tea product provenance. Likewise, the knowledge of tea leaves’ origin is highly valuable to tea producers to assure the quality of the tea being produced. Tea tree (*Camellia sinensis (L.) O. Ktze.*) originated from southwest China, where it has been cultivated for at least 2000 years [1]. Teas (*Camellia sinensis*), which belong to the *Theaceae* family, *Camellia*, are perennial evergreen shrubs or small arbor plants. Interestingly, a “tea seed fossil” more than one million years old was discovered at Yuntou Mountain in Puan County (Guizhou Province, China) [2].

According to 2016 data from the Food and Agriculture Organization (FAO) (http://faostat.fao.org/), the worldwide planting acreage and yield of *Camellia sinensis* are approximately 409.92 × 10^4^ hm^2^ and 5.95 × 10^6^ t, respectively. In China, the *Camellia sinensis* planting acreage is approximately 224.06 × 10^4^ hm^2^ (accounting for 54.66% of the global planted area), and fresh tea leaves production is approximately 2.41 × 10^6^ t (40.56% of the global annual production). Both the planting acreage and yield of *Camellia sinensis* in China ranked first in the world. By the end of 2016, the tea planting area in Guizhou Province (China) was over 4.67 × 10^5^ hm^2^, and the planting area has been ranking the first in China for several years, which shows that Guizhou has been a very important tea production base in China [2]. Its environmental conditions, e.g., low latitude, high altitude, and often cloudy, are ideal for producing high-quality tea [2]. In 2017, green tea produced in Guizhou successfully received the geographical indication (GI) of the Ministry of Agriculture, China, and consequently, GI-certified teas have been sold at much higher prices than noncertified tea products. Anshun (AS), Leishan (LS), Meitan and Fenggang (MTFG) are the main tea trees growing in areas in Guizhou. Unfortunately, fake tea products with fake certificates of GI have appeared on the domestic and international market and have seriously damaged the reputation of Guizhou tea [3,4]. Therefore, the traceability of specific tea is beneficial to protect the brands and GI of Guizhou teas and promote benefits to both consumers and producers.

Many studies have examined the authentication of food and agricultural products, such as onions [5], sechium edule [6], pomelos [7], rice [8,9], winter wheat [10], potatoes [11], wolfberry [12], sesame seeds [13], lemon [14], artichoke [15], olive oils [16], tomato [17], honeysuckle [18], and polygala tenuifolia [19]. In terms of the discrimination of tea leaves origin, a variety of methods have been applied, including high-performance liquid chromatography (HPLC) coupled with spectrophotometry [20], ^1^H nuclear magnetic resonance (^1^HNMR) spectroscopy [21], Fourier transform near-infrared spectroscopy (FT-NIRS) [22], gas chromatography-mass spectrometry (GC-MS) [23], taste sensor techniques [24], and stable isotope signature (C, H, O, N, Sr, and B) technology [3,25]. Among these different analytical methods, inductively coupled plasma mass spectrometry (ICP-MS) and inductively coupled plasma atomic emission spectrometry (ICP-AES) are the most effective and reliable tools for the determination of the quantitative composition of mineral elements in tea leaves. The cost of multielement analysis is lower than that of stable isotopes and organic components. Moreover, the concentrations of mineral elements in tea leaves are also more stable and less affected by the processing steps, storage time, and analytical conditions compared with other components in tea leaves [4,26,27]. For example, Marcos et al. [28] used 26 trace metals to distinguish 15 types of tea from ten different countries. Similarly, 17 trace elements were analyzed to classify tea samples from Asia, Africa, China, India, and Sri Lanka [29]. Ma et al. [4] also analyzed 37 trace elements coupled with LDA to classify the geographical origin of green tea and obtained a high recognition of 98.2% and a satisfactory predictive ability of 96.4%. Moreover, for the white tea from three regions (Fuding city, Zhenghe county, and Jianyang city), the accuracy of origin-based identification using mineral element analyses reached 98.44%, 95.31%, and 100%, respectively by LDA, support vector machines (SVM) analysis, and K-Nearest Neighbor (KNN) methods [30]. Furthermore, Ni et al. [25] reported that 23 elements, 12 metal isotope ratios and four stable isotope ratios (δ^13^C, δ^15^N, δ^2^H, and δ^18^O) were used to discriminate the geographical origin of three types of green tea in combination with PCA, LDA, partial least squares discrimination analysis (PLS-DA), and a decision-making tree (DT), obtaining an accuracy of 90.0% with the DT method.

These studies mainly focused on classifying different categories of tea (green, black, oolong, and scented) or the different countries of origin. However, it is still a great challenge to discriminate the planting regions of tea leaves from the same category (fresh unprocessed leaves) with a small regional scope (Guizhou Province, China). To our knowledge, few studies have been conducted on geographical origin discrimination of Guizhou tea leaves compared to other regions, especially with the application of OPLS-DA [2,25]. It is of great value and meaningful for the global tea trade to trace the geographical origins of Guizhou tea, protect the regional brand and establish a quality safety traceability system for Guizhou tea, as Guizhou is the largest tea raw material center in China.

Accordingly, the aim of the current study was to assess the potential use of multivariate statistics tools (PCA, LDA, and OPLS-DA) combined with multielement data obtained by ICP-MS and ICP-AES analyses of tea leaves for the differentiation of teas according to their geographical origin. This work is different from others reporting the classification of tea sourced from regions separated by large distances, where significant differences in soil composition might be expected and had a remarkable impact on the chemical composition of tea leaves. Finally, this is the first work reporting the classification of tea leaves grown in Guizhou province according to their geographic origin.

## 2. Results and Discussion

### 2.1. Differences in the Elemental Concentrations

The concentrations of 40 mineral elements in tea leaves are summarized in Table 1. The ranges of elements concentrations were in good agreement with the levels obtained in some previous studies [1,3,4]. Especially, the mean concentrations of Mn showed a distributing characteristic of AS > MTFG > LS. Similarly, green tea was found to be a rich source of Mn [31]. A Kolmogorov-Smirnov test showed that the concentrations of the 40 mineral elements in the 87 tea samples followed a normal distribution (*p* > 0.05). Moreover, the average concentrations of Cd, As and Cr in tea samples from these three regions were below the limit standard values (1 mg·kg^−1^, 2 mg·kg^−1^, and 5.0 mg·kg^−1^) of Cd, As, and Cr in NY659-2003 [32]. The Pb concentrations were far below the Chinese National Standard Limit GB2762-2017 (5 mg·kg^−1^) [33]. In addition, the concentration of Cu in the tea leaves from the three regions were lower than the green food (tea) standard limiting value (30 mg·kg^−1^) of the NY/T288-2012 [34]. Besides, compared with the limits (3.0 mg·kg^−1^ for Pb and 1.0 mg·kg^−1^ for Cd) of the law of the European Union [35,36], all of tea leaves samples were below the limit values, which revealed that the quality of the tea leaves in these three regions (Guizhou Province, China) was excellent, and there is no heavy metal pollution in tea leaves from these three regions. Similarly, the concentrations of Pb, Cu, As, Hg, Cd, and Cr in young tea leaves and mature tea leaves from another region (Puan county) of Guizhou Province (China) were all lower than the standard limit values in China [2]. Furthermore, potassium was the most abundant element found in the tea leaves samples overall.

According to the results of one-way ANOVA testing, the mean concentrations of Al, Cu, Fe, Gd, Mg, Mn, Rb, Se, Sm, Sr, Tl, and Zn were not significantly different among the three regions (AS, LS, and MTFG) (*p* > 0.05). Therefore, these twelve elements were rejected for further statistical analysis to discriminate the geographical origin of tea. In contrast, the mean concentrations of 28 elements (As, Ba, Bi, Ca, Cd, Ce, Co, Cr, Cs, Dy, Er, Ho, K, La, Li, Na, Nb, Nd, Ni, P, Pb, Pr, S, Sb, Th, U, W, and Y) in tea leaves were significantly different among the three regions (*p* < 0.05) and could be used for further statistical analysis of traceability. In addition, the Duncan’s multiple comparison analysis was performed for the 28 elements with significant differences (*p* < 0.05). The results showed that the tea leaves from different geographical origins had their own elemental fingerprinting characteristics. In particular, the tea samples from AS exhibited the highest concentrations of As, Bi, Ca, Cr, Cs, Dy, Er, Ho, Li, Nb, Ni, Pb, Th, U, W, and Y and the lowest concentrations of Cd, K, and Na compared with those from the other two regions (LS and MTFG). The tea samples from LS could be clearly differentiated from the other samples based on their highest Ba, Na, and Sb concentrations and the lowest As, Bi, Ca, Ce, Co, Cr, Dy, Er, Th, U, W, Y, Ho, La, Li, Nb, Nd, Ni, P, Pr, and S contents. Moreover, the tea samples from MTFG could be distinguished from the ones from AS and LS as they presented the highest concentrations of S, Cd, Ce, Co, K, La, Nd, P, and Pr and the lowest concentrations of Ba, Cs, Pb, and Sb. Furthermore, the large standard deviations shown by some elements reflected the great variability among samples from the same region, which is similar to the study by Ma et al. [4]. The reason may be that the collected varieties and the elemental compositions of soils were different, which affected elemental concentrations in the tea leaves [8,27].

Different soil conditions in two different tea gardens may be an important factor resulting in great differences in the mineral element content, and soil conditions are also a widely accepted basis for the geographical origins discrimination of tea [30]. However, the influence of tea varieties, picking season, leaves grade of maturity, and other factors cannot be ignored in mineral elements of various geographical regions. In addition, other reports have described the relevance between tea minerals and soil minerals. Zhao et al. [27] proposed that Na, Mg, Ca, Cr, Fe Ni, Rb, Sr, Cd, Tl, and Pb were extremely significant correlated between tea leaves and topsoil at the level of *p* < 0.01. Chen et al. [37] found that the total rare earth element content in soil is positively correlated with that in fresh tea leaves. Zhang et al. [2] reported the concentrations of Mn, Al, Cu, Ni, As and Hg in young tea leaves were positively correlated with the contents of corresponding elements in soil. Nevertheless, it must be noted that the uptake of elements by plants is not solely from the soil but also other possible sources. Notably, a significant correlation of Pb concentrations between young tea leaves and air were found, and surface contamination, caused by atmospheric deposition during tea growth, was the main source for the Pb accumulated in Longjing tea, and the non-edaphic contribution of atmospheric deposition was distance dependent on the proximity to the main traffic area [38]. Conclusively, although there are other possible sources for elements in tea leaves with the exception of soil, regional differences among these three regions can still exist. Thereby, the significant differences in the elemental concentrations in tea leaves provided reliable materials for further statistical analysis of origin discrimination.

### 2.2. Principal Component Analysis (PCA)

Principal component analysis (PCA) is a very important tool, especially in the preliminary steps of multivariate analysis, for performing exploratory analysis to obtain an overview of data and find patterns in complex experimental data [8]. To evaluate the differences in the tea leaves from AS, LS and MTFG, the concentrations of 28 mineral elements with significant differences (*p* < 0.05) were subjected to PCA, an unsupervised technique. Under unsupervised PCA learning, six principal components (PCs) with eigenvalues exceeding one were extracted, which accounted for 75.802% of the total variance. To be specific, each PC is a linear combination of the 28 original measured variables (concentrations of 28 elements). More specifically, PC1, PC2, PC3, PC4, PC5, and PC6 contributed to approximately 32.238%, 18.086%, 9.688%, 6.505%, 5.076%, and 4.210% of the total variance, respectively. The concentrations of Bi, Ca, Ce, Cr, Dy, Er, Ho, La, Li, Nb, Nd, Ni, Pr, Th, U, and Y showed the highest weights in PC1. The concentrations of As, Co, Sb, and Pb had the highest weights in PC2. Additionally, the contents of Cd, Cs, K, P, and S had the highest weights in PC3. The concentrations of Na, W, and Ba were the dominating features variables corresponding to PC4, PC5, and PC6, respectively.

**Figure 1:**
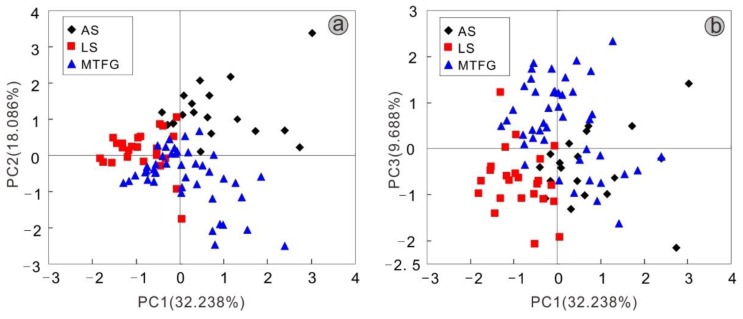
Score scatter plots of the first three principal components (**a**) PC1 versus PC2; (**b**) PC1 versus PC3).

Figure 1 shows the two-dimensional scatter plot of the scores obtained for PC1-PC2 and PC1-PC3, explaining 50.324% and 41.926% of the total variance, respectively. The results indicate that the trends for the cluster of tea samples from the three regions were not very satisfactory and that a partial overlap phenomenon existed among the three groups (Figure 1a). The scatter plot for the PC1-PC3 scores showed the strong overlap among the three groups (AS, LS, and MTFG) (Figure 1b), and the reason could be the lower variance contribution rate. Similar strong overlap results were achieved in studies focused on tea and *Agaricus bisporus* mushroom traceability based on PCA [25,39]. Likewise, the PCA could not distinguish rice samples grown in Korea and China, with the exception of rice samples grown in the Philippines and other regions [8]. Therefore, other statistical analysis methods should be applied to gain better tea classification results.

### 2.3. Linear Discriminant Analysis (LDA)

**Figure 2:**
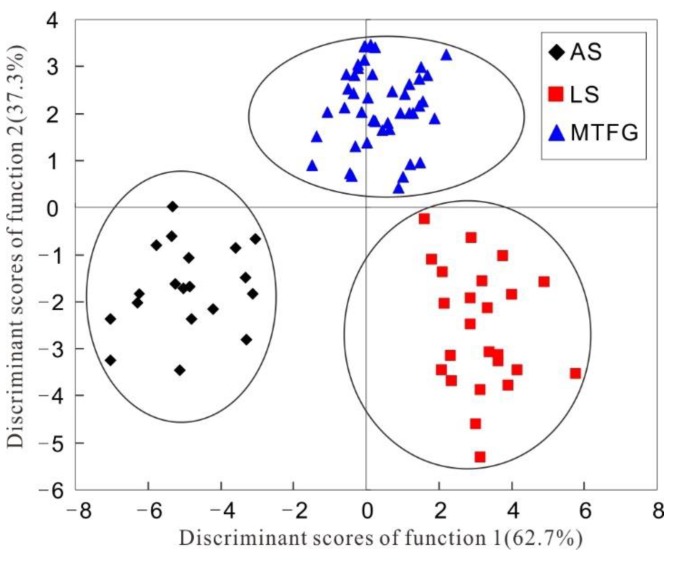
Scores scatter plot of the constructed standardized discriminant functions (function 1 and function 2) of the linear discriminant analysis (LDA) model based on the significantly different elements concentrations.

The linear discriminant analysis technique is a supervised pattern recognition method. In supervised pattern recognition, objects are classified into group clusters with predetermined models for the class. This approach differs from unsupervised methods such as cluster analysis (CA) and PCA [40]. Some researchers have already reported satisfying classification results obtained by LDA [40,41]. To acquire a more satisfying classification of the tea samples originating from the three regions (AS, LS, and MTFG), LDA was performed based on the concentrations of the 28 elements exhibiting significant differences (*p* < 0.05) in the ANOVA test. Consequently, nine elements (As, K, La, Na, Nb, Pb, S, Sb, and U) were selected and two discriminant functions were constructed according to the Wilks’ lambda values. Each discriminant function was evaluated for its statistical significance in terms of the Wilks’ Lambda factor. The range of the Wilks’ Lambda factor is from 1.0 (zero discriminatory power) to 0.0 (perfect discrimination) [4,13]. These two functions explained 100% of the total variance (function 1 explained 62.7% of the total variance and function 2 accounted for 37.3%) (Figure 2), and the Wilks’ lambda values for function 1 (canonical correlation = 0.946) and function 2 (canonical correlation = 0.913) were 0.018 and 0.166, respectively (p_1_ = 0.000 and p_2_ = 0.000, respectively). The results showed that there were significant differences among these three regions. The constructed standardized discriminant functions are as follows:

Function 1 = − 0.492 As + 0.888 K + 0.368 La + 0.563 Na + 0.100 Nb − 0.880 Pb − 0.875 S + 0.591 Sb − 0.036 U
Function 2 = 0.246 As + 0.366 K − 0.011 La − 0.365 Na + 0.593 Nb − 0.924 Pb + 0.148 S − 0.648 Sb + 0.625 U

The discrimination of tea samples from AS, LS, and MTFG was verified by plotting a scatter plot of the two functions’ scores (Figure 2). From Figure 2, it appears that the distribution area of the Meitan and Fenggang (MTFG) tea samples was relatively concentrated. However, the Leishan (LS) tea samples were spread out and thus appeared to be more diverse. Overall, these results indicated that the tea leaves samples from different regions were well distinguished from one another. Thereby, the LDA method based on the mineral element fingerprints can be suitable to verify the origins of the tea leaves.

The discriminant functions achieved a good recognition ability (% of the objects belonging to the training set correctly classified) of 98.9% based on the 28 mineral element concentrations presenting significant differences (*p* < 0.05) (Table 2). To evaluate the predictive capacity, the generated model was validated by the leave-one-out cross-validation method. As a result, the predictive ability of this model (% of the objects belonging to the testing set correctly classified using the developed model) was 98.9% as well, which revealed that the LDA model showed satisfactory results for the classification of tea samples from different regions in Guizhou Province (China). In this LDA, the established Fisher’s linear discrimination functions for each region were as follows:

Group 1 (Anshun) = 175.669 As − 0.001 K − 23.971 La − 0.152 Na − 984.548 Nb + 69.683 Pb + 0.071 S − 291.927 Sb − 3695.292 U − 115.915
Group 2 (Leishan) = 10.517 As + 0.003 K + 27.5 La + 0.162 Na − 813.812 Nb + 6.788 Pb + 0.041 S + 264.153 Sb − 4417.603 U − 94.021
Group 3 (Meitan and Fenggang) = 110.136 As + 0.002 K + 9.008 La − 0.05 Na + 718.577 Nb − 12.620 Pb + 0.054 S − 225.517 Sb − 1800.63 U − 110.986

A very high percentage of correct classification represents the stability and the strong relationship between the extracted profiles and the origins. A very high predictive percentage of the validated set indicated the high ability to classify the origin of the tea samples based on the relative profiles of the selected nine elements (As, K, La, Na, Nb, Pb, S, Sb, and U). Na and Pb were also used to classify the geographical origin of tea in the study by Zhao et al. [27]. Likewise, a high geographical origin discrimination rate of tomato samples (99.9% of cases correctly classified) was acquired according to LDA in the study by Fragni et al. [42]. As a whole, these results indicated that the combination of multi-elemental concentrations and LDA can be a robust and reliable method for geographical origin discrimination of tea samples.

### 2.4. Orthogonal Partial Least Squares Discriminant Analysis (OPLS-DA)

**Figure 3:**
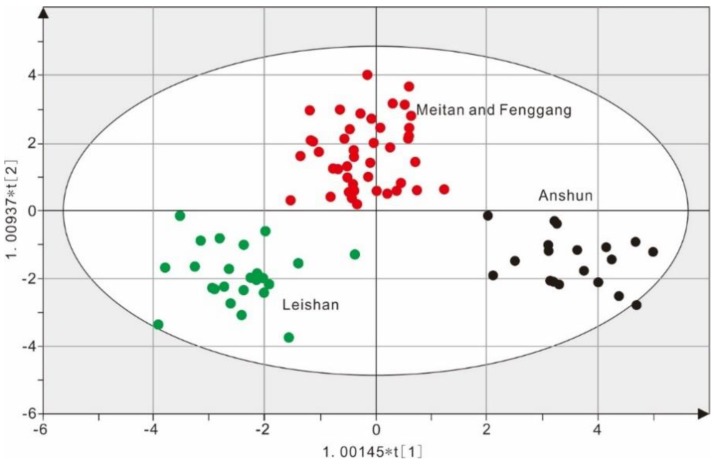
Scores scatter plot of principal components 1 and 2 of the orthogonal partial least squares discriminant analysis (OPLS-DA) results obtained from data on 28 multielement compositions of tea samples with three different geographical origins (Black representing Anshun, Green representing Leishan, and Red representing Meitan and Fenggang). The ellipse on the score plot represents the 95% confidence interval for Hotelling’s T^2^.

OPLS-DA was developed based on partial least squares discriminant analysis (PLS-DA). Compared with PLS-DA, the systematic variation in the X variable is decomposed into two parts, namely, the part linearly related to the Y axis and the part orthogonal to the Y axis. With the increase in the orthogonal variation components, OPLS-DA provides more explanation and reduces the error associated with results [43]. The OPLS-DA performed on the concentrations from 28 elements aimed to differentiate eighty-seven tea samples from the three regions in Guizhou. The results showed that two prediction principal components and two orthogonal principal components were selected from the model constructed by the training set (87 samples). Moreover, for the model parameters, R^2^X = 0.657 showed that the four principal components have a 65.70% explanatory power for the variation in the X variables (31.40% for the predict principal component and 34.30% for the orthogonal principal component). R^2^Y = 0.834 indicated that the two predictive principal components of the model have an explaining ability of 83.40% for the variation in the Y variables. Q^2^ = 0.789 denoted that the two prediction principal components for the model had a prediction ability of 78.90% for tea samples from the three areas (AS, LS, and MTFG). In summary, the constructed OPLS-DA model performed well in this study (Figure 3).

The two-dimensional scatter plot of scores for the first and second principal components is shown in Figure 3. There was an obvious tendency of aggregation for tea samples from each region, and the tea samples from the three tea areas can be well separated from one another. Among them, the X axis was the first principal component and showed that the first principal component can separate the tea samples from the three regions from one another. The Y axis was the second principal component, indicating that the tea samples from MTFG can be separated from AS and LS via the second principal component.

**Figure 4:**
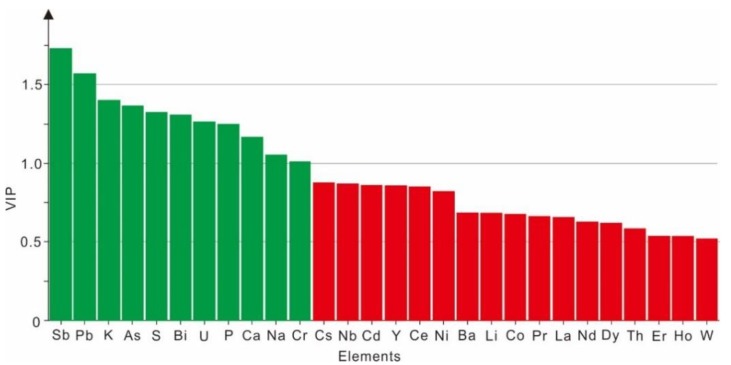
Variable importance in the projection (VIP) obtained from the OPLS-DA model for geographical origin discrimination of tea leaves.

The eighty-seven samples in the training set were used as validation set samples to back test the model to identify the correct classification rate (Table 2). The results are summarized in Table 2. The percentage of correctly classified tea samples using the OPLS-DA model for the three regions was 100%, indicating a very satisfactory classification result. The variable importance in the projection (VIP) is a weighted sum of squares of the PLS weight, and a value >1 is generally used as a criterion to identify a variable’s importance to the model [8,44]. The variable importance in the projection (VIP) values for the OPLS-DA model are shown in Figure 4. Eleven elements (Sb, Pb, K, As, S, Bi, U, P, Ca, Na, and Cr) were found to be significant for determining the geographical origin of the tea samples as a result of their VIP values of >1 (Figure 4). In particular, the VIP values of Sb and Cr were 1.730 (the biggest) and 1.011 (the smallest), respectively, suggesting that Sb can be considered the most significant parameter for geographical origin discrimination among the three tea cultivation regions in this study. Similarly, Pb, K, Bi, Ca, and Cr were found to be significant for differentiating the geographical origin of rice grains from China, the Philippines, and Korea [8]. Moreover, our research results are similar to previous studies [8,11,25,39,45,46] that indicated that multivariate analysis, particularly PLS-DA and OPLS-DA approaches, could clearly distinguish various agricultural products (potatoes, mushrooms, ginseng, cereals, and tea). Furthermore, it was also found that the discriminant accuracy of the OPLS-DA model (completely correct classification, 100%) was higher than the LDA model (existing one mistake sample, 98.9%), demonstrating that the OPLS-DA model is more reliable for discriminating tea leaves from the three regions in Guizhou Province (Table 2).

The results from previous works revealed that different agricultural product cultivars had influences on the correct geographical origin identification rate [47,48]. Therefore, in subsequent research, single tea tree cultivar should be considered for geographical origin traceability of Guizhou tea. It was also confirmed that some stable isotope indicators (δ^13^C, δ^15^N, δ^18^O, δ^2^H, δ^11^B, and ^87^Sr/^86^Sr) could be effective and suitable chemical markers for the regional discrimination of tea samples based on multivariate approaches [3,19,49]. Hence, stable isotope ratios of tea samples, such as δ^13^C, δ^15^N, δ^18^O, δ^2^H, δ^11^B, and ^87^Sr/^86^Sr, can be analyzed to identify new geographical authentication indicators for the regional discrimination of Guizhou tea. Meanwhile, although the results presented in this study indicated that classification can be successful, a complementary study involving a larger number of tea samples would be necessary to establish better classification models for tea samples. Also, other chemometrics tools e.g., k-nearest neighbors (k-NN), support vector machine (SVM) and Random Forest (RF) were applied to classify grapes, which indicated that k-NN, RF and SVM perform best with up to 85.7%, 98.3% and 93.3% accuracy rate, respectively [50]. As a result, we can use these chemometrics tools to discriminate Guizhou tea in future studies.

## 3. Materials and Methods

### 3.1. Sample Collection

**Figure 5:**
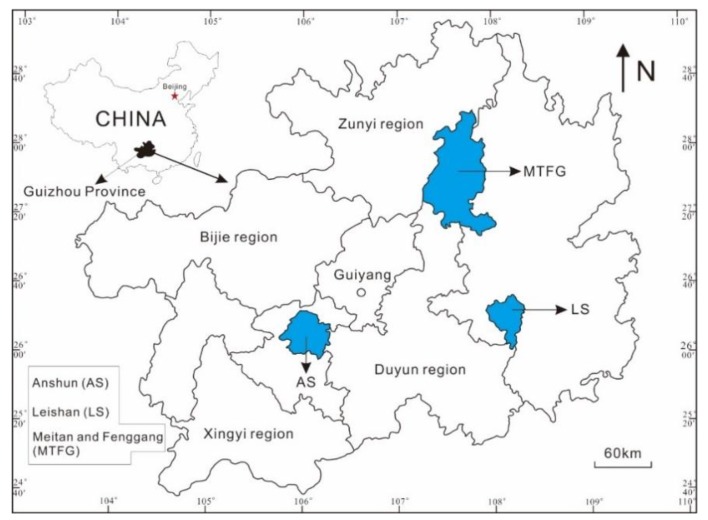
Leaf samples collected from three tea production regions in Guizhou Province, China.

Fresh tea leaf samples (approximately 0.25 kg each) with one bud and two recently expanded leaves were collected from three regions in Guizhou Province (southwest of China), including 19 samples from Anshun City (AS), 24 samples from Leishan County (LS), and 44 samples from Meitan and Fenggang Counties (MTFG) (Figure 5). The longitude and latitude of each sampling site were recorded using a global positioning system (GPS). The tea tree cultivars, including Fuding small-leaf tea, Qianmei 601 tea, and Maitan moss tea, were widely distributed in these three regions. The major soil types in these tea production areas are sandy yellow soil from the weathering of sandstone and mudstone in LS, clayey yellow soils from the weathering of dolomite and limestone in AS, and a mixture of clayey yellow soils and sandy yellow soils in MTFG.

### 3.2. Sample preparation

Leaf samples were washed with tap water and then ultrapure water (18.2 MΩ·cm) to remove the dust and particles on the surface of the leaves. The samples were dried in a thermostatic air-blower-driven drying oven (Langgan Laboratory Equipment Co. Ltd., Shanghai, China) at 60 °C until reaching a constant weight. Subsequently, the tea samples were ground using a portable high-speed universal grinder (60–300 mesh, SE-750, Shengxiang Electrical Appliance Co. Ltd., Yongkang city, Zhejiang Province, China), sieved with a 0.075 mm nylon mesh, and then placed in polyethylene plastic bags for further chemical analysis.

### 3.3. Chemical Analysis

The ground and sieved tea samples were analyzed at an accredited laboratory (ALS Minerals-ALS Chemex Co. Ltd., Guangzhou, China) using ICP-AES (Vista-MPX, Agilent, Santa Clara, CA, USA) and ICP-MS (7700x, Agilent). For the analysis, the ICP-MS and ICP-AES operation parameters proposed by Zhang et al. [2] were used in this study. For the tea sample analysis, a 1.0 g ground and sieved tea sample was accurately weighed, combined with 5 mL concentrated HNO_3_ in a Teflon digestion vessel and digested slowly at room temperature for approximately 8 h to digest the organic matter and release gases before heating for 3 h in a graphite furnace. After cooling, the sample was dissolved and transferred to a volumetric flask and the digestion solution was diluted to a constant volume (25 mL) with 2% hydrochloric acid (HCl).

### 3.4. Quality Control

For quality assurance and quality control (QA/QC) of this analytical procedure, method blanks, duplicates and certified reference materials (CRMs) (GL03 and SpL01) were analyzed. These two standards materials are in-house and self-developed standards and have been used for more than 20 years in ALS Minerals-ALS Chemex. SPL01 is spinifex grass leaves collected from a location near our lab at Mount Isa, Gulf Country of Queensland, Australia. GL03 is made from eucalyptus leaves collected from trees near the lab here in Brisbane, Queensland, Australia. In particular, the CRMs (GL03 and SpL01) were measured 5 times and 8 times, respectively. Nine duplicate samples were set up in this measurement and accounted for 10.3% of the total samples. At least one blank sample was prepared for every ten tea samples in the same manner as described for tea sample digestion. Additionally, the measured values of all the elements in the blank samples were less than or equal to two-fold of the limit of detection (LOD). The LOD, the limit of quantitation (LOQ) (the upper limit of detection), CRM recoveries and relative deviation of duplicate samples (RDDS) for this analytical method are summarized in Table 3.

### 3.5. Statistical Analysis

Data were expressed on the basis of a dry weight (DW) of tea leaves. The Statistical analysis was performed with IBM SPSS Statistics 20.0 (SPSS, Inc., Chicago, IL, USA) and SIMCA-P version 13.0 (Umetrics, Umeå, Sweden). One-way analysis of variance (ANOVA) was used to test the differences of multielement concentrations among the three regions. The differences between each of the two groups were tested by the least significant difference method (LSD). The least-significant difference test was performed at the 0.05 probability level. PCA was used to provide an unsupervised analysis to discriminate the tea samples from three regions using 28 significantly different elements (*p* < 0.05). The dataset with all the significantly different variables was assessed using LDA and OPLS-DA to construct the tea geographical discrimination models. The statistical significance of each discriminant function was evaluated on the basis of the Wilks’ Lambda factor [4]. Predictive ability of the LDA model was evaluated by leave-one-out cross-validation. Furthermore, the S-plot was generated from the OPLS-DA model in order to screen the variables with the largest influence contributing to the separation among groups. Q^2^ is defined as the proportion of variance in the data predicted by a model.

## 4. Conclusions

The concentrations of 28 mineral elements (As, Ba, Bi, Ca, Cd, Ce, Co, Cr, Cs, Dy, Er, Ho, K, La, Li, Na, Nb, Nd, Ni, P, Pb, Pr, S, Sb, Th, U, W, and Y) were significantly different in the three regions (AS, LS, and MTFG) in Guizhou Province (*p* < 0.05). The tea samples from these three regions can be well separated from one another based on the twenty-eight significantly different elements coupled with LDA and OPLS-DA models. Moreover, classifying results from the OPLS-DA model (correct classification of 100%) performed better than the LDA model (98.9%). Eleven elements (Sb, Pb, K, As, S, Bi, U, P, Ca, Na, and Cr) were regarded as good tracers for geographical origin identification of tea samples in the OPLS-DA. Overall, the combination of mineral element fingerprinting and chemometrics can be an effective and reliable method for the geographical origin discrimination of tea from different regions.

## Figures and Tables

**Table 1:** Minimum (Min), maximum (Max), average concentrations (Mean), standard deviations (SD) of concentrations of 40 elements in tea leaves collected from Anshun (AS), Leishan (LS), and Meitan and Fengggang (MTFG).

Element	AS (n = 19)				LS (n = 24)				MTFG (n = 44)				*p*-Value
	Min	Max	Mean	SD	Min	Max	Mean	SD	Min	Max	Mean	SD	
Al ^b^	270	540	371	72	240	530	416	78	200	630	420	100	0.126 ^c^
As ^b^	0.073	0.221	0.128	0.039	0.020	0.112	0.050	0.024	0.028	0.099	0.059	0.018	0.000 ^d^
Ba ^b^	7.3	31.2	12.7	5.6	7.4	19.7	12.8	3.5	2.7	28.4	9.7	5.1	0.018 ^d^
Bi ^b^	0.006	0.022	0.013	0.004	0.002	0.010	0.005	0.002	0.002	0.013	0.006	0.002	0.000 ^d^
Ca ^b^	0.29	0.52	0.39	0.07	0.20	0.31	0.25	0.03	0.22	0.42	0.31	0.05	0.000 ^d^
Cd ^b^	0.028	0.088	0.051	0.019	0.016	0.243	0.053	0.046	0.022	0.180	0.081	0.034	0.001 ^d^
Ce ^b^	0.071	0.345	0.156	0.082	0.037	0.162	0.096	0.038	0.047	0.828	0.215	0.163	0.001 ^d^
Co ^b^	0.068	1.530	0.419	0.443	0.065	0.676	0.320	0.171	0.152	2.610	0.628	0.530	0.019 ^d^
Cr ^b^	0.49	1.85	0.91	0.35	0.13	0.73	0.41	0.19	0.19	1.16	0.53	0.25	0.000 ^d^
Cs ^b^	0.090	1.740	0.480	0.438	0.052	0.592	0.220	0.148	0.018	0.752	0.162	0.179	0.000 ^d^
Cu ^b^	15.10	26.10	18.50	2.55	10.80	24.10	17.28	3.36	11.40	22.10	16.94	2.35	0.115 ^c^
Dy ^a^	4.5	23.9	10.2	5.6	1.9	22.6	6.0	4.5	3.0	24.5	9.7	5.4	0.011 ^d^
Er ^a^	2.4	17.8	5.8	3.5	0.8	13.9	3.1	3.2	1.3	17.6	5.2	3.6	0.028 ^d^
Fe ^b^	57	275	115	50	66	189	111	28	83	173	114	20	0.934 ^c^
Gd ^a^	5.0	35.7	12.4	7.7	2.1	28.5	8.2	6.2	4.3	27.9	12.0	6.8	0.065 ^c^
Ho ^a^	0.8	6.4	2.0	1.4	0.1	4.9	1.1	1.0	0.4	4.9	1.8	1.1	0.025 ^d^
K ^b^	1.38	2.29	1.63	0.21	1.63	2.23	1.94	0.17	1.72	2.39	2.06	0.16	0.000 ^d^
La ^b^	0.036	0.181	0.079	0.044	0.016	0.178	0.059	0.039	0.029	0.295	0.100	0.070	0.021 ^d^
Li ^b^	0.05	0.22	0.10	0.04	0.02	0.13	0.06	0.03	0.04	0.17	0.08	0.03	0.001 ^d^
Mg ^b^	0.15	0.28	0.19	0.03	0.15	0.23	0.19	0.02	0.15	0.24	0.20	0.02	0.185 ^c^
Mn ^b^	159	2050	921	570	273	1555	743	331	205	2290	836	450	0.436 ^c^
Na ^b^	20	70	47	13	30	100	73	19	30	80	52	15	0.000 ^d^
Nb ^a^	2.6	12.2	5.3	2.3	0.5	5.4	2.6	1.3	1.3	7.8	3.6	1.5	0.000 ^d^
Nd ^b^	0.022	0.149	0.057	0.034	0.009	0.122	0.041	0.029	0.021	0.188	0.067	0.043	0.027 ^d^
Ni ^b^	11.55	34.50	18.78	6.28	5.55	19.65	11.58	4.03	6.63	33.00	13.24	4.88	0.000 ^d^
P ^b^	0.476	0.620	0.541	0.040	0.406	0.551	0.482	0.042	0.427	0.639	0.559	0.049	0.000 ^d^
Pb ^b^	0.413	0.937	0.574	0.153	0.106	0.476	0.268	0.092	0.093	0.463	0.218	0.073	0.000 ^d^
Pr ^b^	0.006	0.037	0.015	0.008	0.003	0.035	0.011	0.008	0.006	0.053	0.019	0.012	0.019 ^d^
Rb ^b^	16.7	83.0	53.0	19.0	21.7	135.0	66.8	25.2	16.7	161.0	53.0	31.3	0.116 ^c^
S ^b^	0.271	0.390	0.322	0.029	0.252	0.316	0.289	0.017	0.282	0.383	0.333	0.024	0.000 ^d^
Sb ^b^	0.021	0.070	0.038	0.010	0.022	0.071	0.045	0.014	0.009	0.032	0.019	0.006	0.000 ^d^
Se ^b^	0.07	0.18	0.11	0.03	0.04	0.17	0.09	0.03	0.05	0.21	0.11	0.04	0.114 ^c^
Sm ^b^	0.003	0.034	0.011	0.007	0.002	0.025	0.008	0.006	0.003	0.040	0.013	0.009	0.054 ^c^
Sr ^b^	5.28	12.85	7.89	2.23	2.51	11.15	5.88	1.81	3.30	18.60	6.92	3.21	0.055 ^c^
Th ^b^	0.003	0.037	0.008	0.007	0.002	0.010	0.005	0.002	0.002	0.012	0.006	0.002	0.006 ^d^
Tl ^b^	0.009	0.044	0.024	0.012	0.003	0.030	0.012	0.006	0.004	0.139	0.021	0.022	0.058 ^c^
U ^a^	2.8	8.6	4.8	1.5	1.0	4.3	2.3	0.7	1.8	6.6	4.4	1.2	0.000 ^d^
W ^b^	0.010	0.235	0.060	0.069	0.007	0.193	0.033	0.037	0.011	0.096	0.033	0.018	0.033 ^d^
Y ^b^	0.033	0.191	0.074	0.045	0.008	0.113	0.032	0.024	0.024	0.174	0.067	0.033	0.000 ^d^
Zn ^b^	43.8	62.2	52.4	5.0	41.1	59.8	48.3	5.5	40.6	75.7	50.9	6.0	0.058 ^c^

^a^ The unit for the element concentrations was µg·kg^−1^. ^b^ The unit for the element concentrations was mg·kg^−1^. ^c^ There is no significant difference for the element concentrations among the three regions (*p* > 0.05). ^d^ There is significant difference for the element concentrations among the three regions (*p* < 0.05). The units of Ca, K, Mg, P, and S were %. Data were expressed on the basis of a dry weight (DW) of tea leaves.

**Table 2:** Classification results of tea samples and percentage of observations correctly classified by LDA and OPLS-DA based on 28 significantly different elements of 87 tea samples from the three regions.

Tea region	Model	Verification Samples	Predicted Group Membership			Correctly Classified (%)
			AS	LS	MTFG	
AS	LDA	19	19	0	0	100
LS		24	0	23	1	95.8
MTFG		44	0	0	44	100
Total		87	19	23	45	98.9
AS	OPLS-DA	19	19	0	0	100
LS		24	0	24	0	100
MTFG		44	0	0	44	100
Total		87	19	24	44	100

**Table 3:** The limits of detection (LOD), limit of quantitation (LOQ), recoveries of certified reference materials (CRMs), and relative deviation of duplicate samples (RDDS).

Elements	LOD (μg·g^−1^)	LOQ (μg·g^−1^)	Recoveries of CRMs (%)	RDDS (%)
Al	10	25,000	77.78−128.57	0.00−7.84
As	0.005	10,000	88.74−108.53	1.35−18.75
Ba	0.1	9000	87.88−103.03	0.00−2.13
Bi	0.001	9000	83.33−117.39	0.00−33.33
Ca	10	40,000	93.25−109.68	0.00−3.85
Cd	0.001	2000	85.71−128.57	0.00−9.23
Ce	0.001	500	90.46−105.88	0.72−6.90
Co	0.002	9000	91.12−109.52	0.17−5.64
Cr	0.05	10,000	90.13−113.92	2.00−34.18
Cs	0.001	500	95.00−115.00	0.84−12.50
Cu	0.01	9000	92.34−109.91	0.23−5.61
Dy	0.0005	1000	85.00−110.00	2.08−23.53
Er	0.0005	1000	81.40−125.00	0.00−17.24
Fe	1	50,000	89.76−109.34	0.38−5.22
Gd	0.0005	1000	84.92−103.70	0.70−17.33
Ho	0.0001	1000	87.50−106.25	0.00−33.33
K	10	100,000	93.68−108.84	0.27−2.20
La	0.001	9000	90.65−107.41	0.00−8.33
Li	0.02	10,000	86.67−113.33	0.00−11.11
Mg	10	300,000	91.11−110.34	0.23−2.36
Mn	0.1	50,000	91.05−110.31	0.45−3.09
Na	10	100,000	80.00−120.00	0.00−25.00
Nb	0.0005	500	82.50−112.80	3.23−20.00
Nd	0.001	1000	84.14−105.00	0.00−18.52
Ni	0.02	9000	92.44−109.00	0.00−5.57
P	5	50,000	93.22−108.55	0.21−2.52
Pb	0.005	9000	89.05−109.52	0.11−7.64
Pr	0.001	1000	83.33−112.90	0.00−11.11
Rb	0.01	9000	97.99−108.43	0.39−8.14
S	10	100,000	93.10−110.10	0.00−2.48
Sb	0.002	9000	82.47−107.10	1.82−22.08
Se	0.02	1000	96.23−117.39	0.00−12.50
Sm	0.001	1000	82.61−114.81	0.00−25.00
Sr	0.02	10,000	96.49−107.84	0.25−6.32
Th	0.001	1000	87.50−106.45	0.00−23.08
Tl	0.001	1000	94.87−115.38	0.00−5.75
U	0.0005	9000	87.97−112.78	0.00−32.08
W	0.002	9000	86.21−115.25	0.00−20.55
Y	0.001	500	89.55−110.45	0.65−8.40
Zn	0.1	9000	93.62−109.12	0.20−5.23
